# Psychometric Evaluation of the Cross‐Culturally Adapted Dutch‐Language Version of the Participation and Environment Measure for Children and Youth (PEM‐CY)

**DOI:** 10.1155/oti/5537119

**Published:** 2026-03-20

**Authors:** Eefje Kern, Barbara Piškur, Sarah Meuser, Rachel Teplicky

**Affiliations:** ^1^ Faculty of Healthcare and Wellbeing, Academy of Occupational Therapy, Zuyd University of Applied Sciences, Heerlen, the Netherlands, zuyd.nl; ^2^ Faculty of Healthcare and Wellbeing, Research Centre for Autonomy and Participation, Zuyd University of Applied Sciences, Heerlen, the Netherlands, zuyd.nl; ^3^ Faculty of Rehabilitation Sciences, REVAL Rehabilitation Research Center, Hasselt University, Diepenbeek, Belgium, uhasselt.be; ^4^ Faculty of Health, Medicine and Life Sciences (SHE), Maastricht University, Maastricht, the Netherlands, maastrichtuniversity.nl; ^5^ CanChild Centre for Childhood Disability Research, McMaster University, Hamilton, Ontario, Canada, mcmaster.ca

## Abstract

**Background:**

Participation in meaningful activities is essential for children′s development and well‐being, making it a key focus of rehabilitation outcomes. The PEM‐CY assesses participation frequency, involvement, and environmental support in home, school, and community settings. While it has been validated in several languages, its psychometric properties have not yet been evaluated in the Dutch context.

**Objectives:**

Therefore, the aim of our study is to assess the internal consistency, test–retest reliability, and construct validity of the Dutch PEM‐CY including its ability to differentiate between children with and without disabilities and to examine associations between environmental supportiveness, participation, and parental satisfaction.

**Methods:**

A cross‐sectional study involving parents of 161 Dutch children 5–17 years old with and without disabilities was conducted to psychometrically evaluate the Dutch PEM‐CY in the Netherlands. Internal consistency was evaluated using Cronbach′s alpha, test–retest reliability with intraclass correlation coefficients (ICCs), and construct validity through known‐group comparisons and correlations between environmental supportiveness, participation outcomes, and parental desire for change.

**Results:**

The Dutch PEM‐CY demonstrated acceptable to good internal consistency (*α* = 0.54–0.68) and good to very good test–retest reliability (ICC = 0.60–0.80). Known‐group comparisons revealed significant differences between children with and without disabilities, particularly in involvement and environmental barriers. In addition, higher perceived environmental support was significantly associated with greater involvement and less parental desire for change, but not with participation frequency. These findings support the Dutch PEM‐CY′s reliability and validity in assessing participation and environmental influences among Dutch children aged 5–17 years old.

**Conclusion:**

The Dutch PEM‐CY is a reliable and valid instrument for assessing participation and environmental influences among Dutch children aged 5–17 years. Its ability to identify both group differences and associations with environmental support underscores its value for guiding tailored occupational therapy interventions and informing inclusive policy development.

## 1. Introduction

Research has indicated that children with a disability participate less frequently in daily activities and feel more isolated than children without disabilities [1–4]. Over the last two decades, enabling children′s participation in all kinds of life situations has received increasing attention and is seen as an important outcome of rehabilitation [5]. Participation in life situations means that a child attends by being present in the participatory context and also feels involved, a subjective experience of participation in the moment that includes affect, motivation, persistence, and social connection [6, 7].

The literature has shown that the environment can either support or limit the participation of children with a disability [8–12]. For instance, physically inaccessible spaces can limit the participation of children and youth with disabilities [13–15], whereas physically accessible or accommodating facilities could be seen as enablers for children′s participation [16, 17]. Additionally, others′ negative attitudes and lack of information have been identified as possible hindering factors for participation [13, 14]. Furthermore, a supportive social environment, such as parents, peers, teachers, community members, and friends, has been shown to be essential [18]. In particular, parents greatly influence participation at school, at home, and in the community [19, 20]. Research has shown that parents undertake many actions to improve their children′s participation in daily life and they can be seen as experts in enabling their child′s participation [21]. Consequently, to enhance the participation of children with disabilities and improve pediatric rehabilitation services, strong collaboration between healthcare professionals and parents is essential, allowing parents to share their knowledge and combine efforts. This need for collaboration aligns with current societal changes, emphasizing partnerships with parents, shared decision‐making, empowerment, and cross‐sector collaboration between care, education, and well‐being services [22]. Because the developmental therapy process to enhance participation begins with assessing the current situation, it is crucial to gather information about the child′s participation across various environments and the impact of these environments. At this early stage, parents together with the child should be the primary source of information.

Since the launch of the International Classification of Functioning, Disability and Health [6], an increasing number of measures of participation have been developed and adopted across the globe, such as the Children′s Assessment of Participation and Enjoyment (CAPE) and the Preferences for Activities of Children (PAC) [5, 23–25]. However, only a few measures have addressed the influence of the environment on a child′s participation; one is the Participation and Environment Measure for Children and Youth (PEM‐CY) developed by the PEM‐CY CanChild research team [4]. The PEM‐CY is the first measure that combines parental evaluation of children′s participation in home, school, and community activities with an assessment of the environmental factors that either facilitate or hinder participation [26]. The PEM‐CY stands out from broader environmental measurement tools, such as the Craig Hospital Inventory of Environmental Factors (CHIEF) for Children–Parent Version [27], by focusing on environmental influences on participation rather than solely on child‐specific factors. To our knowledge, none of the Dutch measures used to assess participation in pediatric rehabilitation or other settings addresses environmental factors in the same way as they are defined and formulated in the PEM‐CY. The use of the PEM‐CY in the Dutch context of pediatric rehabilitation and/or education could enable professionals to give parents a voice and to gain a more holistic picture of factors that influence children′s participation. However, the direct application of assessment instruments developed in other regions is often inappropriate. Simple translation is insufficient, as participation is inherently context‐dependent and culturally constructed [7]. Factors such as physical infrastructure (e.g., cycling culture and public transport), the organization of the educational system, and prevailing social norms regarding disability substantially shape how participation is enacted and perceived, particularly in comparison with North American contexts [28]. According to the International Test Commission [29], foreign assessment tools must undergo a careful and often time‐consuming adaptation process before they can be effectively used in a different context [30–32].

**Figure 1:**
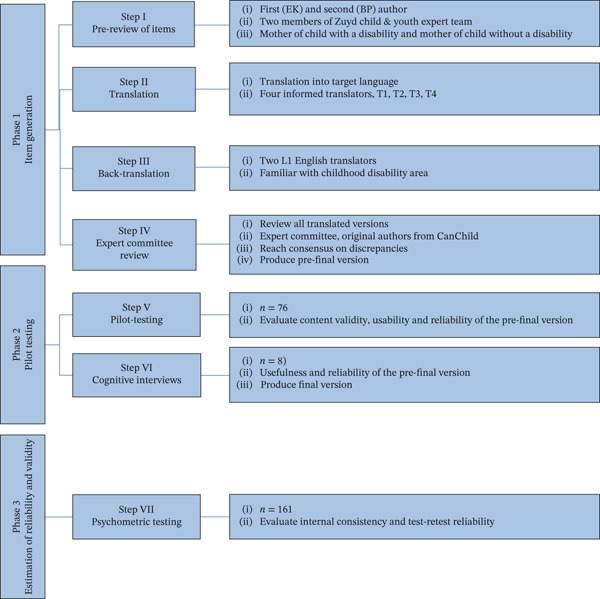
Graphical representation of the methodology applied in the process of translation and cultural adaptation of the PEM‐CY for the Dutch language, adapted from Beaton et al. [33].

Prior to this study, the PEM‐CY was translated and cross‐culturally adapted for use in the Netherlands in collaboration with CanChild′s PEM‐CY research team, using the procedure proposed by Beaton et al. [33], following Steps I–IV (see Figure 1). In Phase 1, the authors (E.K. and B.P.) and the Zuyd Child and Youth Expert team (content experts) translated the PEM‐CY into Dutch. After evaluation and consensus on technical and cultural validity, this version was piloted with end users (mothers of children with and without disabilities) (Steps I–II). The translation was then back‐translated into English by two independent bilingual individuals (Step III). After corrections by the content experts, the prefinal version was discussed with the CanChild PEM‐CY team to reach consensus on any discrepancies (Step IV). Then, in Phase 2, the prefinal Dutch version of the PEM‐CY was pilot‐tested with a small target population (*n* = 76) to evaluate its content validity in the Dutch context (Step V). Face validity, content validity, efficiency, and appropriateness were assessed [34]. Although the pilot showed good efficiency, appropriateness, acceptable internal consistency, and test–retest reliability, content validity was found to be inadequate. To address this, an additional qualitative study [35] was conducted, using a cognitive interview approach with eight parents (six with children with disabilities and two with children without disabilities; Step VI). This study led to several recommended changes concerning (1) choosing a similar semantic expression (e.g., “neighborhood” instead of “community”), (2) an extra response option, and (3) structural changes in the layout to improve the visual structure. These changes were incorporated into the revised version of the Dutch PEM‐CY. This version was then prepared for psychometric testing, as the measurement properties of the PEM‐CY have not yet been tested in the Dutch context. The aim of this study is to psychometrically evaluate the Dutch‐language version of the PEM‐CY. Based on the original Canadian validation study [36] and in accordance with COSMIN standards, specific research questions and corresponding a priori hypotheses were formulated:

1. Do scores on the PEM‐CY (Dutch) identify differences in participation and perceived impact of the environment (a) between children with and without disabilities and (b) across age groups?

Hypothesis: We expect scores to differ significantly (a) between children with and without disabilities (lower scores for the disability group) and (b) across age groups.

2. Is there a relation between perceived supportiveness of the environment and (a) parents′ satisfaction with (or desire for change in) their child′s participation and (b) participation frequency?

Hypothesis: We expect environmental support to be positively related to (a) parents′ satisfaction (i.e., less desire for change) and (b) participation frequency.

## 2. Methods

### 2.1. Study Design and Ethical Approval

A quantitative, cross‐sectional study design was used in this study to examine the psychometric properties of the PEM‐CY (Dutch). The study was reviewed and approved by the Atrium‐Orbis‐Zuyd Ethics Committee (No. 15‐N‐51). The study was conducted in accordance with the principles of the Declaration of Helsinki (2013). All parents provided written informed consent prior to participation.

### 2.2. Instrument

The PEM‐CY is a parent‐report survey measure, developed in Canada and the United States [4] that evaluates (a) child′s participation across a broad range of home, school, and community‐based activities; (b) multiple dimensions of a child′s participation (frequency, involvement, change desired); and (c) parent′s perception of the impact of a broad range of environmental factors (e.g., physical layout; social, cognitive demands of activities) on the child′s participation in a specific setting (i.e., home, school, and community) and includes (d) relevant content and response options for parents of youth with and without a disability. A list of summary scores for the PEM‐CY [4] is presented in Table 1. The PEM‐CY was initially designed and validated for use in population‐based research studies involving children 5–17 years old with and without disabilities. The original version of the PEM‐CY [36] showed moderate to very good internal consistency (*α* > 0.59) and moderate to good test–retest reliability (ICC = 0.58–0.95). The PEM‐CY can also detect significant differences in the participation profiles of children with and without disabilities in the home, school, and community, even when controlling for the child′s age, gender, and/or annual household income [2, 37–39].

**Table 1:** Participation and environment measure for children and youth summary scores.

Section	Description	Summary score
Participation		
Frequency (%)	Provides an indicator of the frequency of engagement in activities in that setting	Sum of all ratings except “never” divided by the number of ratings
Participates ever (%)	Provides an indicator of the range of activities the child participates in	Number of items answered with frequency other than “never” divided by the total number of items rated
Involvement	Extent to which the child is engaged in activities, including social engagement	Average level across activities (average of all items except those to which parents responded “never” for frequency)
Desire for change (%)	Indirect indicator of satisfaction with current participation	Number of “yes, change” responses divided by the total number of items rated
Environment		
Resources (%)	Overall indicator of the extent to which the family perceives it has adequate resources to support the child′s participation	Sum of resource item ratings divided by the number of items rated
Supports (%)	Overall indicator of the extent to which the environment is perceived to support the child′s participation	Sum of support item ratings divided by the number of items rated
Supportiveness, total (%)	Global indicator of the extent to which the environment supports participation in that setting	Sum of ratings divided by the number of items rated

### 2.3. Participants

Parents of children between 5 and 17 years old with and without disabilities participated in the study (Phase 3). A sample size of at least 100 participants is recommended by the COSMIN standards to ensure adequate power for evaluating internal consistency and reliability [31]. Therefore, we aimed for a sample size exceeding 100 participants. The convenience sample consisted of 161 parents recruited in the Limburg region using a gatekeeper approach. Parents of children with and without disability were recruited by occupational therapists (gatekeepers) from occupational therapy practices who provided services for children with disabilities and from primary schools. Inclusion criteria were (a) being a parent or legal guardian of the child between 5 and 17 years old (with or without a disability) at the time of enrolment and (b) the ability to speak and/or read Dutch language.

### 2.4. Procedure

Gatekeepers distributed information about this study to parents, including a consent form, research description, demographic questionnaire, and a paper version of the Dutch PEM‐CY. A short demographic assessment was created for this study to ensure diversity in child age, disability, schooling situation, and parent education level. Participants received a written information sheet detailing the project and signed a consent form agreeing to participate. Participants who consented to the test–retest component of the study were invited to complete the PEM‐CY survey a second time within 4 weeks of the initial administration. The survey was distributed by mail and included a prepaid return envelope to facilitate completion and return. A reminder was sent by mail during this period. To protect privacy, all research materials were assigned an ID code for anonymity and confidentiality and stored in the Zuyd data repository.

### 2.5. Data Analysis

Statistical analyses were conducted using SPSS Statistics 19 data analysis software. Means and standard deviations, frequencies, and percentages were used to describe continuous variables. Internal consistency was examined by evaluating the extent to which the Dutch PEM‐CY items measure the same attributes. Internal consistency for the “participation frequency,” “involvement,” and “desire for change” subscales, as well as the “environment” subscales, was investigated by calculating Cronbach′s *α*. In this study, we expected that the internal consistency of most scales would be moderate (0.70 ≤ *α* ≤ 0.85). This expectation aligns with findings from Coster et al. [36], which recognize that multiple factors, such as individual preferences, family values, and priorities, can influence the overall pattern of participation in activities within a particular context. Test–retest reliability was assessed by calculating the ICC consistency (two‐way random effects model) to investigate whether the summary scores on the PEM‐CY are reproducible across occasions. The minimum acceptable level for test–retest reliability was set at 0.70.

Effects of disability on participation were examined using the Mann–Whitney *U* test. Age group differences in PEM‐CY summary scores were analyzed using the Kruskal–Wallis test, and ordered patterns across age groups were assessed with the Jonckheere–Terpstra test. For these nonparametric group comparisons, effect sizes were calculated as *r* (*r* = *z*/√*N*).

Additionally, associations between perceived environmental supportiveness, parents′ satisfaction with their child′s participation, and level of involvement were examined using Kendall′s tau correlation coefficient (*p* < 0.05), whereas *r* quantified the effect size of group differences in nonparametric tests.

## 3. Results

### 3.1. Participant Demographics

A total of 161 parents or guardians of children with and without a disability participated in this study. Thirty‐two of these participants agreed to participate in the test–retest phase of the study and provided adequate data for analysis. Not all participants completed all sections of the questionnaire; therefore, the number of participants included in the analysis for different contexts (home, school, and community) varied due to missing values. The majority of respondents were mothers (88%), and 65% of the children with disabilities were male. Most children with and without disabilities were younger than 12 years old with the “with disability” group consisting of 77 children (M_age_ = 8.7 years, SD = 2.642) and the “without disability” group consisting of 84 children (M_age_ = 9.4 years, SD = 3.517). The most common primary functional impairments or diagnoses reported by parents were developmental delay (40.3%) and autism spectrum disorder (15.6%). For a more detailed presentation of the descriptive characteristics of the parents and their children, see Table 2.

**Table 2:** Participant characteristics.

	Parent of a child with disabilities*n* (%)77 (48)	Parent of a child without disabilities*n* (%)84 (52)
Respondent relation with child		
Mother	68 (88)	70 (83)
Father	5 (6)	13 (16)
Guardian	4 (6)	1 (1)
Respondent age (years)		
20–29	0 (0)	2 (3)
30–39	30 (39)	33 (39)
40–49	44 (57)	42 (50)
50–59	3 (4)	6 (7)
Other	0 (0)	1 (1)
Respondent education		
High school	2 (3)	1 (1)
Community college	25 (32)	27 (32)
University of Applied Sciences	40 (52)	41 (49)
University	10 (13)	14 (17)
Other	0 (0)	1 (1)
Child sex		
Male	50 (65)	48 (57)
Female	27 (35)	36 (43)
Child age (years)		
5–8	43 (56)	39 (47)
9–11	18 (24)	17 (20)
12–14	13 (17)	19 (23)
15–17	2 (3)	8 (10)
Younger than 12	61 (80)	56 (68)
12 and older	15 (20)	27 (32)
Child education		
Mainstream primary education	45 (58)	59 (70)
Special primary education	24 (31)	0 (0)
Mainstream secondary education	6 (8)	24 (29)
Special secondary education	2 (3)	1 (1)
Primary functional impairment/diagnosis		
Developmental delay	31 (40.3)	
Intellectual delay	10 (13)	
Hearing impairment	2 (2.6)	
Speech/language impairment	4 (5.2)	
Vision impairment	0 (0)	
Emotional impairment	2 (2.6)	
Orthopedic impairment	2 (2.6)	
Autism spectrum disorder	12 (15.6)	
Multiple disabilities	4 (5.2)	
Not reported, not known (other)	10 (13)	

### 3.2. Internal Consistency and Test–Retest Reliability

The internal consistency coefficients were 0.59, 0.55, and 0.54 for “participation frequency” and 0.68, 0.65, and 0.60 for “involvement,” across home, school, and community, respectively. Internal consistency coefficients for the environment scales were ≥ 0.70 for all but two scales: “home support” (0.69) and “home resources” (0.68). Since the Cronbach′s test uses listwise deletion for missing values, the number of participants included varied in this analysis. Analysis of the test–retest reliability for the participation and environment scales yielded good to very good ICC values ranging between 0.60 and 0.88 in all settings, except the “participates ever” and “involvement” scales in the school and community settings, which showed moderate ICCs ranging from 0.50 to 0.57. Cronbach′s *α* and ICC values are shown in Table 3.

**Table 3:** Internal consistency (*α*) and test–retest reliability (intraclass correlation).

Variable	Cronbach′s alpha						Intraclass correlation		
	Home		School		Community		Home	School	Community
		*n*		*n*		*n*	*n* = 31–32	*n* = 31–32	*n* = 31–32
Participation									
Frequency	0.59	158	0.55	158	0.54	157	0.68	0.65	0.60
Participates ever							0.71	0.50	0.57
Involvement	0.68	150	0.65	139	0.60	130	0.75	0.57	0.51
Environment									
Supportiveness	0.78	145	0.82	154	0.84	156	0.82	0.85	0.88
Support	0.69	147	0.80	157	0.82	160	0.81	0.85	0.88
Resources	0.68	157	0.74	156	0.70	157	0.70	0.78	0.75

### 3.3. Construct Validity

#### 3.3.1. Differences in Participation for Children With and Without Disabilities and Across Age Groups

According to parent reports, children with a disability showed similar participation frequency (“participation frequency”) and activity variety (“participates ever”) in the *home* setting compared to peers without a disability (medians of 6.1 vs. 6.2 and 90 vs. 100, respectively). These differences were not statistically significant (*U* = 2668.5, *p* = 0.09, *r* = −0.13; effect size calculated as *r* = *z*/√*N*), suggesting comparable frequency and variety of home participation across groups. However, children with a disability showed significantly lower involvement in these home activities compared to their peers without a disability (*U* = 2248.5, *p* = 0.00, *r* = −0.25). Further analysis revealed significant differences in the range of activities children engaged in at home across different age groups (*H*(3) = 3, *p* = 0.00, *r* = 0.26).

This trend held when we separated children with and without disabilities; *H*(*p*) < 0.02. Interestingly, children with disabilities showed an increase in the range of activities participated in across the first three age groups, followed by a significant decrease in the oldest age group; *J*(*p*) = 0.02 (see Table 4).

**Table 4:** Comparison of “participation” and “environment”—Home scores, by disability and age group.

Home variables	Disability groupMean (SD)		*U*	*p*	Effect size (*r*)	Age groupMean (SD)				*H* (*p*)	*J* (*p*)	*z*	Effect size (*r*)
	Yes	No				5–8 years	9–11 years	12–14 years	15–17 years				
Participation													
Frequency	6.1 (0.49)	6.2 (0.43)	2668.5	0.09	−0.13	6.0 (0.5)	6.2 (0.5)	6.2 (0.4)	6.3 (0.6)	3 (0.04)	4875 (0.00)	2.90	0.23
Participates ever (%)	90 (13.1)	100 (16.9)	2842	0.16	−0.11	87.8 (12.4)	90.5 (19.1)	96.9 (5.4)	88 (11.4)	3 (0.00)	4987.5 (0.00)	3.25	0.26
Involvement	3.9 (0.54)	4 (0.51)	2248.5	0.00	−0.25	3.8 (0.6)	3.9 (0.5)	4.1 (0.5)	4.1 (0.5)	3 (0.02)	4884 (0.00)	2.94	0.23
Desire for change (%)	40 (25.7)	30 (24.5)	2507.5	0.02	−0.19	36.6 (24.7)	35.7 (27.3)	47.2 (25.8)	41 (16)	3 (0.25)	4472.5 (0.17)	1.38	0.11
Environment													
Support (%)	85.7 (10.4)	100 (4.88)	1140	0.00	−0.57	91.4 (9.7)	88.1 (10)	92.7 (10)	96.2 (5.4)	3 (0.04)	4242.5 (0.39)	0.85	0.07
Resources (%)	93.3 (10.1)	100 (4.73)	2368	0.00	−0.26	95.2 (9.2)	95.2 (7.5)	96.2 (5.3)	94.7 (8.2)	3 (0.99)	4023 (0.91)	−0.11	−0.01
Supportiveness total (%)	91.7 (9.0)	97.2 (3.8)	1267.5	0.00	−0.53	92.9 (8.4)	91.3 (8.1)	94.2 (7.3)	95.3 (5.9)	3 (0.18)	4266 (0.48)	0.71	0.06

*Note:* Disability group differences tested with Mann–Whitney *U*; *U* = test statistic, effect size *r* from *z*. Age group differences tested with Kruskal–Wallis (*H*) and Jonckheere–Terpstra (*J*). *p* values indicate the significance of group differences/trends. Disability group sample size range: disability, *n* = 77; no disability, *n* = 82–84. Age group sample size ranges: 158–159; Ages 5–8, *n* = 82; Ages 9–11, *n* = 34–35; Ages 12–14, *n* = 32; Ages 15–17, *n* = 10.

In the *school* setting, children with a disability were significantly less involved in activities at school than children without a disability (*U* = 2216, *p* = 0.00, *r* = −0.23). However, no significant differences in involvement were observed across different age groups (see Table 5).

**Table 5:** Comparison of “participation” and “environment”—School scores, by disability and age group.

School variables	Disability groupMean (SD)		*U*	*p*	Effect size (*r*)	Age groupMean (SD)				*H* (*p*)	*J* (*p*)	*z*	Effect size (*r*)
	Yes	No				5–8 years	9–11 years	12–14 years	15–17 years				
Participation													
Frequency (%)	5.0 (0.9)	4.8 (0.8)	2864.5	0.38	−0.06	4.8 (0.9)	5.0 (0.9)	4.7 (0.9)	4.7 (0.7)	3 (0.58)	3681.5 (0.57)	−0.57	−0.05
Participates ever (%)	80 (21)	80 (18.6)	2712.5	0.06	−0.15	80.5 (17.5)	84 (18)	76.3 (25.1)	72 (27)	3 (0.28)	3911 (0.62)	−0.50	−0.04
Involvement	4.2 (0.8)	4.5 (0.6)	2216	0.00	−0.23	4.2 (0.6)	4.1 (0.9)	4.4 (0.7)	4.3 (0.7)	3 (0.52)	4165.5 (0.18)	1.33	0.11
Desire for change (%)	20 (27.5)	0 (21.9)	2180	0.02	−0.27	25.5 (26.6)	22.3 (26.4)	17.4 (22)	20 (26.7)	3 (0.56)	3519.5 (0.17)	−1.39	−0.11
Environment													
Support (%)	81.5 (11.3)	96.3 (7.2)	1209	0.00	−0.54	88.8 (10.2)	86.5 (12.3)	89.4 (13.1)	95.4 (4.9)	3 (0.19)	4282.5 (0.26)	1.12	0.09
Resources (%)	95.8 (11.6)	100 (3.6)	2192.5	0.00	−0.31	96 (7.6)	91.7 (11.9)	95.2 (8.9)	98.8 (2.8)	3 (0.04)	3975.5 (0.78)	−0.28	−0.02
Supportiveness total (%)	88.2 (9.4)	98 (5)	1130.5	0.00	−0.57	92.3 (7.7)	89 (9.8)	91.7 (10.5)	97 (2.6)	3 (0.07)	4306.5 (0.40)	0.83	0.07

*Note:* Disability group differences tested with Mann–Whitney *U*; *U* = test statistic, effect size *r* from *z*. Age group differences tested with Kruskal–Wallis (*H*) and Jonckheere–Terpstra (*J*). *p* values indicate the significance of group differences/trends. Disability group sample size range: disability, *n* = 74–77; no disability, *n* = 82–84. Age group sample size range: 154–159; Ages 5–8, *n* = 79–82; Ages 9–11, *n* = 34–35; Ages 12–14, *n* = 30–32; Ages 15–17, *n* = 9–10.

In the *community* setting, children with disabilities participated in a narrower range of activities (“participates ever”) compared to children without disabilities (*p* = 0.00). Interestingly, both groups showed a significant increase in the variety of activities from the youngest age group to subsequent age groups (*J* = 4660.5, *p* = 0.05, *z* = 2.01, *r* = 0.16). Additionally, the frequency of participation differed significantly by age group and increased consistently with age for the entire sample (see Table 6). Subgroup analyses suggest that children with disabilities aged 12 years and older may participate less frequently and in fewer activities compared to same‐age peers, although these data are not presented.

**Table 6:** Comparison of “participation” and “environment”—Community scores, by disability and age group.

Community variables	Disability groupMean (SD)		*U*	*p*	Effect size (*r*)	Age groupMean (SD)				*H* (*p*)	*J* (*p*)	*z*	Effect size (*r*)
	Yes	No				5–8 years	9–11 years	12–14 years	15–17 years				
Participation													
Frequency (%)	4.4 (0.9)	4.5 (0.8)	3163.5	0.81	−0.02	4.5 (0.8)	4.4 (0.6)	3.8 (0.9)	4.6 (0.8)	3 (0.00)	3197 (0.01)	−2.78	−0.22
Participates ever (%)	60 (17.2)	70 (15.1)	2361	0.00	−0.23	60.4 (17)	67.4 (12.7)	65 (19.3)	66 (15.1)	3 (0.15)	4660.5 (0.05)	2.01	0.16
Involvement	4.3 (0.7)	4.3 (0.5)	3109	0.67	−0.03	4.2 (0.6)	4.1 (0.7)	4.2 (0.6)	4.3 (0.5)	3 (0.78)	3911 (0.65)	−0.46	−0.04
Desire for change (%)	20 (23.9)	10 (17.3)	2452.5	0.01	−0.20	22.7 (22.5)	18.8 (17.7)	23.8 (22.4)	10 (13.3)	3 (0.25)	3772.5 (0.47)	−0.73	−0.06
Environment													
Support (%)	88.9 (12.8)	100 (7.8)	1896	0.00	−0.37	90.6 (9.5)	86.2 (14.8)	94.1 (10.1)	93 (11.5)	3 (0.05)	4503 (0.13)	1.51	0.12
Resources (%)	100 (10.6)	100 (8.6)	2819.5	0.12	−0.12	94.6 (9.1)	93.5 (9.1)	94.7 (10.3)	90 (14.5)	3 (0.54)	3998 (0.84)	−0.20	−0.02
Supportiveness total (%)	91.7 (10.3)	97.9 (7.3)	2120.5	0.00	−0.30	92.3 (7.8)	89.4 (10.8)	94.4 (9.6)	91.6 (12.8)	3 (0.08)	4405.5 (0.24)	1.17	0.09

*Note:* Disability group differences tested with Mann–Whitney *U*; *U* = test statistic, effect size *r* from *z*. Age group differences tested with Kruskal–Wallis (*H*) and Jonckheere–Terpstra (*J*). *p* values indicate the significance of group differences/trends. Disability group sample size range: disability, *n* = 76–77; no disability, *n* = 82–84. Age group sample size range: 158–159; Ages 5–8, *n* = 82; Ages 9–11, *n* = 34–35; Ages 12–14, *n* = 32; Ages 15–17, *n* = 10.

#### 3.3.2. Differences in Perceived Impact of the Environment for Children With and Without Disabilities

Parents of children with disabilities reported significantly more environmental factors restricting their child′s participation across all settings (home, school, and community) compared to parents of children without disabilities. This pattern was consistent for support, resources, and overall environmental supportiveness in each setting. In the *home* environment, disability was associated with lower reported environmental support, resources, and overall supportiveness (*U* = 1267.5, *p* = 0.00, *r* = −0.53). Children with disabilities faced significantly more restricting factors compared to their peers without disabilities, particularly regarding the child′s relationships with others, as well as the social, cognitive, and physical demands of activities (see Table 4). Similarly, in the *school* environment, disability was associated with lower reported support (*p* < 0.001, *r* = −0.54), resources (*r* = −0.31, *p* < 0.001), and overall environmental supportiveness (*p* < 0.001, *r* = −0.57) (see Table 5). Descriptive analysis revealed that children with disabilities experienced more barriers in the attitudes and actions of teachers or staff, relationships with peers, and the social, cognitive, and physical demands of school activities than children without disabilities. The *community* setting followed the same pattern; parents of children with disabilities reported significantly less support (*U* = 1896, *p* < 0.001, *r* = −0.37) and lower overall environmental supportiveness (*U* = 2120.5, *p* < 0.001, *r* = −0.30) than parents of children without disabilities (see Table 6). The most frequently reported barriers for children with disabilities in community settings were the attitudes and actions of other community members, the child′s relationship with others, and the social, cognitive, and physical demands of activities.

Analysis across settings revealed three consistent restricting factors: the child′s relationship with others, the attitudes and actions of teachers/staff in school settings, and the social, cognitive, and physical demands of activities. These factors emerged as significant barriers in home, school, and community environments. Regarding environmental resources, parents of children with disabilities identified different key restrictions per setting. In the school environment, “policies and procedures,” “programs and services,” and “access to personal and public transportation” were considered the most participation‐restricting factors for children with disabilities. In the community setting, “programs and services,” “information,” and “time investment” were identified as the main participation‐restricting factors by parents of children with disabilities.

#### 3.3.3. Desire for Change

In all settings, parents of children with a disability had a significantly greater desire for change in their child′s participation compared to parents of children without a disability (*p* < 0.05). Parents of children with and without disabilities reported a desired change in participation frequency and participation involvement more than the desire for participation in a greater variety of activities in all settings.

### 3.4. Correlations

#### 3.4.1. Perceived Supportiveness of the Environment and Desire for Change

Results from the total sample indicated an inverse relationship between the extent to which the environment supports participation and the desire for change parents reported across all settings: home, with *τ*(160) = −0.19, *p* ≤ 0.01; school, with *τ*(158) = −0.28, *p* < 0.01; and community, with *τ*(160) = −0.25, *p* < 0.01. These findings suggest that as the supportiveness of the environment increases, the desire for change decreases. This relationship was particularly evident in the community setting for children with disabilities (*τ* = −0.25, *p* < 0.01) indicating that environmental supportiveness may be especially important in reducing parents′ desire for change in their child′s participation in community‐based activities.

#### 3.4.2. Perceived Supportiveness of the Environment and Participation Frequency and Involvement

Overall environmental supportiveness was significantly positively correlated with involvement across settings in the total sample: home, with *τ*(159) = 0.24, *p* < 0.01; school, with *τ*(156) = 0.33, *p* < 0.01; and community, with *τ*(161) = 0.15, *p* < 0.01. For children with disabilities, environmental supportiveness was correlated significantly with involvement in the home (*τ* = 0.18, *p* < 0.05) and school (*τ* = 0.16, *p* < 0.05) settings, but not in the community setting. For children without disabilities, environmental supportiveness was significantly correlated with involvement in the school (*τ* = 0.37, *p* < 0.01) and community (*τ* = 0.17, *p* < 0.05) settings.

In contrast, no significant correlations were found between environmental supportiveness and participation frequency across settings for the total sample or for either of the disability groups. These results suggest that while the supportiveness of the environment appears to enhance the quality of participation (involvement), it does not necessarily increase how often children participate in activities (frequency).

All correlations between environmental factors and participation variables are presented in Table 7.

**Table 7:** Correlations between environmental factors and participation variables.

Variable		Home *τ*			School *τ*			Community *τ*		
		Total sample *n* = 159	With disability *n* = 77	Without disability *n* = 82	Total sample *n* = 155	With disability *n* = 74	Without disability *n* = 82	Total sample *n* = 160	With disa bility *n* = 76	Without disability *n* = 84
Participation frequency	Desire for change	−0.25^∗∗^	−0.26^∗∗^	−0.21^∗∗^	−0.10	−0.14	−0.12	−0.25^∗∗^	−0.33^∗∗^	−0.18^∗^
Participation frequency	Involvement	0.25^∗∗^	0.20^∗∗^	0.26^∗∗^	0.06	0.02	0.15	0.22^∗∗^	0.20^∗∗^	0.25^∗∗^
Involvement	Desire for change	−0.36^∗∗^	−0.38^∗∗^	−0.32^∗∗^	−0.31^∗∗^	−0.30^∗∗^	−0.23^∗∗^	0.37^∗∗^	−0.36^∗∗^	−0.32^∗∗^
Environment support	Involvement	0.25^∗∗^	0.18^∗^	0.15	0.33^∗∗^	0.19^∗^	0.40^∗∗^	0.13^∗^	0.09	0.15
Environment supportiveness	Involvement	0.24^∗∗^	0.18^∗^	0.12	0.33^∗∗^	0.16^∗^	0.37^∗∗^	0.15^∗∗^	0.13	0.17^∗^
Environment supportiveness	Desire for change	−0.19^∗∗^	−0.12	−0.13	−0.28^∗∗^	−0.16	−0.21^∗^	−0.25^∗∗^	−0.25^∗∗^	−0.20^∗^

^∗^Statistically significant at the 0.05 level (two‐tailed).

^∗∗^Statistically significant at the 0.01 level (two‐tailed).

## 4. Discussion

The aim of this study was to psychometrically evaluate the Dutch‐language version of the PEM‐CY. The findings indicate that the Dutch version demonstrates sufficient internal consistency and test–retest reliability, supporting its use in the Netherlands. Furthermore, in line with the research questions, the Dutch version was able to detect expected differences in participation and environmental impact between children with and without disabilities and across age groups, as well as to capture associations between environmental supportiveness, parents′ desire for change, and participation frequency and involvement. These findings are consistent with those of the original PEM‐CY (Cronbach′s *α* = 0.59–0.96; [36]) and with validations in other cultural contexts, including India (*α* = 0.61–0.87; [40]), Brazil (*α* = 0.70–0.95; [41]), China (*α* = 0.55–0.86; [42]), Korea (*α* = 0.67–0.92; [43]), and Turkey (*α* = 0.67–0.80; [44]). The consistency of these psychometric properties across diverse cultures supports the robustness of the PEM‐CY as a global measure.

*Research Question 1*: Differences in participation and perceived impact of the environment between children with and without disabilities and across age groups.

Confirming our first hypothesis, known‐group comparisons showed that the PEM‐CY (Dutch) can effectively detect expected differences in participation and the perceived impact of the environment between children with and without disabilities across all settings and age groups. While participation frequency and variety were similar between children with and without disabilities in certain contexts, children with disabilities consistently showed significantly lower levels of involvement across all settings.

In the home setting, children with disabilities demonstrated similar participation frequency and variety compared to their peers without disabilities, but their level of involvement in activities was significantly lower. This finding aligns with results from Chinese and Korean validation studies [42, 43], which emphasized that children with and without disabilities may participate in comparable ways; however, their engagement in these activities often differs significantly. These findings are also consistent with earlier research indicating that children with disabilities encounter additional challenges in participation and involvement compared to their peers without disabilities [10, 24]. Moreover, studies by Coster et al. [4], King [45], and Imms et al. [7] highlighted a critical distinction between “doing” (participation frequency and variety) and “being engaged” (involvement). This underlines that children with disabilities may participate, but often experience lower levels of meaningful involvement. Addressing both the quantity and quality of participation is therefore essential to support meaningful participation for children with disabilities.

In the school setting, parents of children with disabilities reported significantly less support from the school environment and significantly less involvement by their child in school activities. The cognitive and social demands of an activity, the attitudes of others, and the child′s relationship with peers were mostly reported as barriers. These findings are consistent with the experiences of parents in the study by Piškur et al. [46], who also reported restrictions in school support due to differences in perception regarding a child′s disabilities and a lack of professional know‐how that resulted in too little anticipation of the child′s needs in the classroom and outside. In the present study, 38% of the children who were attending mainstream education had a disability. Inclusion of students with disabilities in regular education programs has increasingly been emphasized in the educational policies in many countries [47]. The Dutch school system was reformed in August 2014, and the new Law of Inclusive Education and Opportunities requiring all children to be included in mainstream education has taken effect [48]. Due to this reform, the lack of knowledge teachers in mainstream education may have about a child′s disability and ways to support the child can have a negative environmental impact on the child′s level of participation involvement/engagement.

In the community setting, children with disabilities participated in a narrower range of activities and reported a lower frequency of participation compared to children without disabilities. Participation in community‐based activities, which often depends on peer interaction and accessible environments, was especially limited for older children with disabilities. Significant age‐related differences in participation were observed, with children without disabilities showing increased participation as they grew older, while participation among children with disabilities declined during adolescence. This finding aligns with previous research indicating this decline [49–52]. For children without disabilities, the increase in activity diversity and participation intensity in the home and community settings is consistent with developmental theory, which suggests that as children mature, they broaden their interests, engage in more activities outside the home, and participate in increasingly social forms of play [53].

The decline in participation for children with disabilities during adolescence may be attributed to increasing social demands, the attitudes of others, and challenges in relationships, which are especially prevalent in community‐based activities. The reported barriers arising from social demands, others′ attitudes, and relationship difficulties may explain why children with disabilities from the age of 12 years participate less frequently in community activities involving social engagement, such as community events, peer groups, and informal gatherings compared to their same‐age peers without disabilities. These findings underscore the importance of creating inclusive environments and addressing social and attitudinal barriers so as to foster sustained participation across all age groups.

*Research Question 2*: Relation between perceived supportiveness of the environment and (a) parents′ satisfaction with (or desire for change in) their child′s participation and (b) participation frequency.

In support of our second hypothesis, this study affirms a significant correlation between environmental supportiveness and participation involvement, as well as environmental supportiveness and parental desire for change. Our findings show an inverse relationship between environmental supportiveness and parents′ desire for change across all settings. This indicates that when the environment is perceived as more supportive, parents report less desire for change in their child′s participation, whereas lower levels of environmental supportiveness are associated with stronger parental desire for change. Interestingly, while environmental supportiveness did not significantly correlate with participation frequency in any setting for children either with or without disabilities, it did show significant positive correlations with involvement across all settings. This distinction highlights that supportive environments may enhance the quality of participation (involvement) rather than simply increasing how often children participate in activities.

These results underscore the importance of addressing environmental barriers to improve the quality of participation and parental satisfaction. The findings suggest that interventions focused on enhancing environmental supportiveness might be more effective in improving meaningful engagement (involvement) rather than merely increasing the frequency of participation.

Comparing the current results globally highlights that environmental factors are highly culture‐dependent. First, the perception of the construct varies; the Brazilian validation [41] noted that parents often prioritize body functions over participation, leading to different “desire for change” scores. Second, differences in society require adaptations. For instance, the Korean study [43] removed items like “working for pay” because they are not common in that culture. Furthermore, the study showed that the specific school system strongly influences participation. Similarly, both the German study [28] and the present study suggest that the transition toward inclusive education creates specific environmental barriers. This confirms that cultural adaptation is necessary to correctly understand the specific situation in each country.

### 4.1. Implications for Future Practice

A good understanding of environmental barriers and supports is essential for developing effective services to increase children′s participation in the home, school, and community settings. Many interventions to date have been focused primarily at the level of the individual child. New developments in education and the healthcare system call for a strategic leveled approach to interventions that, for example, create environments that facilitate participation for all children (e.g., the Partnering for Change [P4C] model; [54]) or influence policies. Healthcare providers such as occupational therapists and physiotherapists may have an active role in being a partner with children and families to build their capacity to modify or adapt the environment for more successful participation. Furthermore, clinicians should be aware that participation is culturally defined. The Dutch PEM‐CY allows therapists to move beyond a “one‐size‐fits‐all” approach and engage in a dialog with parents about what constitutes meaningful participation within their specific family culture and Dutch societal context. They can also support and advocate for involving people with disabilities as end users and experts based on their life experiences when developing policies that will influence their participation. Finally, the PEM‐CY offers a common language for parents and professionals, facilitating a shared understanding to prioritize the needs of the child and family.

We recommend that future research should include children′s unique perspectives on their participation when developing programs and services to effectively meet children′s and young people′s needs. This study also shows that environmental interventions may be even more crucial to support participation than interventions directed at a child′s disabilities and it is important to continue to explore this.

### 4.2. Limitations

The limitations of the study are important considerations when interpreting the results. In this study, participants were selected through purposive sampling by occupational therapists working mostly in primary care. While the sample size was considered sufficient according to COSMIN standards for the specific psychometric properties evaluated in this study (internal consistency and specific hypotheses testing), it was not randomly selected, and it may limit the generalizability of the results to the broader population of children with disabilities in the Netherlands; for example, children attending primary care may have less severe disabilities than children attending occupational therapy services in secondary care (e.g., rehabilitation centers).

The results reported were obtained from data collected using the paper version of the Dutch PEM‐CY, and it is not known if the responses would be the same if the survey was administered digitally using the Internet. The extent and moderate complexity of the PEM‐CY may have influenced the way participants completed the questionnaire. Missing values in the dataset could have been avoided by using a survey tool that does not allow participants to skip questions.

## 5. Conclusion

This study demonstrates that the Dutch PEM‐CY is a reliable and valid tool for assessing participation and environmental support in home, school, and community settings in the Netherlands for children aged 5–17 years, with and without disabilities. The instrument supports clinical assessment by identifying participation restrictions and environmental influences, supporting intervention planning in clinical practice. The findings also highlight the impact of the supportiveness of the environment on participation, particularly for children with disabilities. Lower supportiveness was associated with reduced involvement, emphasizing the need for environment‐based interventions such as PREP [55] and P4C [54]. Future research should explore the implementation of these approaches to enhance participation outcomes.

## Funding

No funding was received for this manuscript.

## Disclosure

This article is a revised and supplemented version of the first author′s master′s thesis.

## Conflicts of Interest

The authors declare no conflicts of interest.

## Data Availability

The data that support the findings of this study are available from the corresponding authors upon reasonable request.
